# Rheological Evolution and Viscoelastic Transition of Ambient-Curing Epoxy–Urethane Reactive Polymer Composites

**DOI:** 10.3390/polym18131581

**Published:** 2026-06-25

**Authors:** Xinmei Zhang, Yan Shi, Dongliang Wang, Biao Ma, Jianmin Liao, Tao Chen

**Affiliations:** 1 Key Laboratory for Special Area Highway Engineering of Ministry of Education, Chang’an University, Xi’an 710064, China; zhangxinmei@chd.edu.cn (X.Z.); shiyan@chd.edu.cn (Y.S.); 2 Shanxi No. 8 Construction Engineering Group Co., Ltd., Taiyuan 030027, China; dl_wang12@163.com; 3 Guangxi Machinery Industry Research Institute Co., Ltd., Nanning 530007, China; liao1357liao@163.com (J.L.); chen7531chen@163.com (T.C.)

**Keywords:** epoxy–urethane composite, ambient curing, rheological evolution, three-interval thixotropy, viscoelastic transition, apparent gel time

## Abstract

Ambient-curing epoxy–urethane reactive polymer composites require a balance between initial flowability and subsequent structure buildup. In this study, epoxy–urethane reactive polymer composites containing precipitated calcium carbonate were prepared and referred to as EUPC formulations. Their rheological evolution was characterized by flow sweep, temperature sweep, time sweep, three-interval thixotropy tests (3ITT), amplitude sweep, and oscillatory time sweep. The formulations exhibited distinct initial flow resistance and strong temperature sensitivity, with apparent viscosity decreasing as temperature increased. During ambient curing, viscosity increased continuously, indicating progressive rheological buildup under the selected testing conditions. The 3ITT results showed high-shear-induced apparent viscosity reduction followed by recovery-stage viscosity evolution after returning to the low-shear condition, indicating that the recovery index should be interpreted as an apparent post-shear recovery index rather than a purely thixotropic recovery parameter. Oscillatory measurements revealed a gradual transition from viscous-dominated to more elastic-dominated behavior, and the apparent gel time followed the sequence EUPC-2 < EUPC-4 < EUPC-1 < EUPC-3 < EUPC-5 < EUPC-6. These results indicate that EUPC processability and structure buildup should be evaluated by integrating initial viscosity, temperature sensitivity, post-shear response, and operational viscous-to-elastic transition.

## 1. Introduction

Ambient-curing reactive polymer composites have attracted increasing attention because they can be processed as flowable uncured systems and subsequently develop internal structure under room-temperature curing conditions [1]. Compared with heat-cured systems, ambient-curing materials are particularly suitable for field repair applications in which external heating, prolonged thermal conditioning, or high-temperature equipment is impractical. Typical applications include pavement crack sealing, localized defect repair, joint filling, and in situ rehabilitation of narrow or irregular defects. In these applications, the uncured material must maintain sufficient initial flowability during mixing and placement to penetrate confined defect spaces, and then progressively develop elasticity, cohesion, and dimensional stability after placement [2,3].

From a rheological perspective, the central challenge is to balance processability with curing-time-dependent rheological buildup [4,5]. Processability is commonly reflected by initial flow resistance and shear-dependent flow behavior, whereas structure buildup is reflected by time-dependent viscosity evolution and the viscous-to-elastic transition. Excessively high initial viscosity can hinder mixing, pumping, and penetration, whereas excessively low viscosity may result in poor shape retention or leakage after placement [3,6,7]. In addition, rapid viscosity buildup can shorten the practical working window, while delayed network development may compromise early structural stability [5,8]. Therefore, ambient-curing reactive composites should be evaluated using coupled flow and viscoelastic indicators rather than relying on a single viscosity or mechanical-strength index [4,9,10,11].

Epoxy-based materials have been widely investigated for pavement repair, sealing, and other adhesive applications because of their high cohesive strength, strong adhesion to mineral substrates, and favorable durability [12,13,14,15]. Epoxy asphalt binders, epoxy asphalt mastics, epoxy-modified mixtures, and related reactive pavement materials have also shown considerable engineering potential [8,16,17,18]. However, neat epoxy networks are usually brittle and may not provide sufficient deformability to accommodate thermal contraction, traffic-induced movement, and localized strain concentration in asphalt pavement systems [16,17,19]. Polyurethane modification provides an effective strategy for improving the flexibility and rheological adaptability of epoxy-based systems [20,21,22]. Polyurethane-containing pavement materials and polyurethane-modified asphalt systems have been reported to exhibit improved elasticity, deformation capacity, and rheological response [23,24,25,26]. In epoxy–urethane systems, flexible polyurethane segments can help reduce the rigidity of the epoxy-rich phase, increase segmental mobility, and reduce internal stress concentration, thereby improving low-temperature deformability while maintaining the advantages of chemical curing and network formation [20,22,27].

Filler incorporation is another important approach for regulating the rheological behavior, dimensional stability, and microstructural development of reactive polymer composites [24,28,29]. Precipitated calcium carbonate (PCC), as a mineral filler, is of particular interest because CaCO_3_-based fillers can influence viscosity, viscoelastic response, toughness, and filler–matrix interactions in polymer composites. In filled reactive systems, PCC may affect viscosity and viscoelastic response through particle–matrix interactions. However, its contribution depends on the surrounding polymer matrix, filler-to-binder ratio, and curing state. Therefore, the role of PCC should be interpreted within the overall formulation system rather than treated as an isolated compositional factor [24,30,31].

Although crack-sealing materials, epoxy-based pavement materials, polyurethane-modified systems, and reactive polymer composites have been extensively studied, the integrated rheological evolution of ambient-curing epoxy–urethane reactive polymer composites remains insufficiently clarified [14,15,32,33]. Previous studies have often emphasized macroscopic adhesion, aging resistance, mechanical strength, or durability, whereas limited attention has been paid to the combined evolution of flowability, temperature sensitivity, time-dependent viscosity buildup, post-shear rheological response, and viscoelastic transition in ambient-curing epoxy–urethane reactive systems [2,34,35,36]. Moreover, studies on gel rheology, gel-point identification, post-shear recovery, and resin-based viscoelastic materials indicate that flow behavior, recovery-stage viscosity evolution, and viscous-to-elastic transition are important factors for designing reactive polymer systems with controllable processing behavior and structural development [4,9,10,11].

In this study, ambient-curing epoxy–urethane reactive polymer composites containing precipitated calcium carbonate as a mineral filler were prepared using E44 epoxy resin, an isocyanate-terminated polyether polyurethane prepolymer, polypropylene glycol diglycidyl ether, trimethylolpropane tris(3-mercaptopropionate), and 2,4,6-tris(dimethylaminomethyl)phenol. These composites are hereafter referred to as EUPC. Flow sweep, temperature sweep, time sweep, three-interval thixotropy test, amplitude sweep, and oscillatory time sweep measurements were conducted to characterize the shear-dependent flow response, temperature-dependent viscosity behavior, time-dependent viscosity evolution, post-shear rheological response, and viscous-to-elastic transition of the uncured EUPC mixtures. In this work, apparent gelation is operationally defined as a rheologically identified viscous-to-elastic transition under the selected oscillatory testing condition, rather than as a strict frequency-independent critical gel point [9,10]. The objectives of this study were to: (1) compare the formulation-dependent rheological responses of EUPC systems under selected testing conditions; (2) evaluate high-shear-induced apparent viscosity reduction and apparent post-shear recovery; and (3) provide a laboratory-scale rheological basis for interpreting the processability–structure buildup balance of ambient-curing epoxy–urethane reactive polymer composites.

## 2. Materials and Methods

### 2.1. Materials, Formulation Design, and Preparation of EUPC

The preparation of EUPC was carried out using the raw materials listed in Table 1.

The formulation proportions are listed in Table 2. The formulation design was established by regulating the dilution level of the reactive matrix, the thiol–epoxy stoichiometric level, and the filler-to-binder ratio while maintaining a constant epoxy–urethane modification level. E44 was fixed at 100 parts by mass, and the E44: PUP mass ratio was kept at 100:25 for all formulations. PPGDGE was introduced at three dosage levels to adjust the viscosity and flexibility of the reactive matrix. TMPMP dosage was determined according to the designed thiol–epoxy stoichiometric level, and PCC was incorporated according to two nominal filler-to-binder ratios. This design generated six formulations with different initial flow resistance and curing-related rheological evolution, enabling comparative evaluation of the processability–structure buildup balance of EUPC systems.

**Figure 1:**
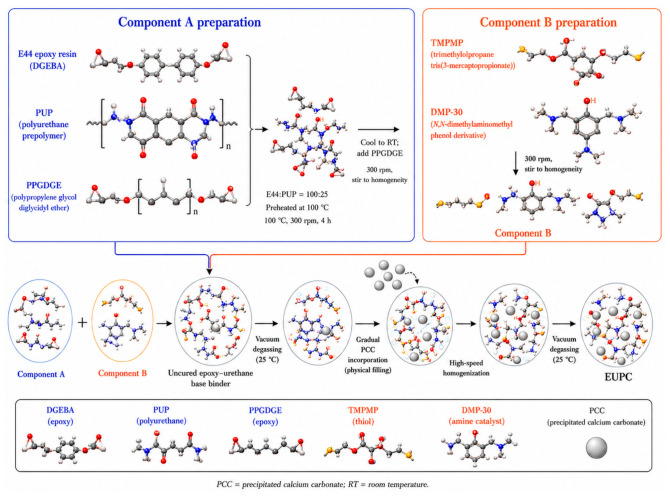
Preparation and pre-curing structural arrangement of EUPC reactive polymer composites.

For sample preparation, Component A was prepared by mixing DGEBA, PUP, and PPGDGE, whereas Component B was prepared by mixing TMPMP and DMP-30. Components A and B were then combined to form the uncured reactive binder. PCC was subsequently incorporated and dispersed into the binder, followed by vacuum degassing before rheological testing or curing. The preparation route is illustrated in Figure 1.

Figure 1 schematically illustrates the preparation sequence and the expected arrangement of the main organic and inorganic components before curing. The schematic is intended to describe the formulation and mixing process.

### 2.2. FTIR and DSC Characterization

Fourier transform infrared spectroscopy (FTIR) was used to qualitatively identify characteristic functional groups in the raw materials and cured EUPC formulations and to compare representative changes in absorption bands after curing [37]. FTIR spectra were recorded using an IRPrestige-21 spectrometer (Shimadzu, Kyoto, Japan) over a wavenumber range of 4000–500 cm^−1^. Absorption bands associated with epoxy, mercapto, urethane, carbonyl, C–O, and O–H groups were assigned for qualitative structural characterization. The FTIR results were used for qualitative comparison of functional-group changes rather than for quantitative evaluation of curing conversion [38,39].

Differential scanning calorimetry (DSC) was used to evaluate the thermal transition behavior of the cured EUPC formulations. DSC measurements were performed using a DSC 200 F3 differential scanning calorimeter (NETZSCH, Selb, Germany) under a nitrogen atmosphere. Approximately 5–10 mg of each cured sample was sealed in an aluminum pan and heated from −50 to 100 °C at a heating rate of 5 °C/min. The glass transition temperature, Tg, was determined as the midpoint of the baseline shift in the DSC thermogram.

### 2.3. Rheological Measurements

Rheological measurements were performed using a Discovery HR-20 hybrid rheometer (TA Instruments, New Castle, DE, USA) equipped with a 25 mm parallel-plate geometry. The testing gap was fixed at 2.0 mm, and the temperature control accuracy was ±0.2 °C. Freshly prepared EUPC samples were loaded immediately after preparation to minimize uncontrolled curing before measurement. During loading, the sample was placed at the center of the lower plate, and excess material was carefully trimmed after setting the gap to ensure stable contact and a smooth sample edge. Before each test, the sample was subjected to pre-shear at 100 s^−1^ for 60 s, followed by a 60 s rest period. The preset test program was then started immediately. The plate surfaces were thoroughly cleaned between consecutive tests to avoid cross-contamination.

Flow sweep tests were conducted to characterize the shear-rate-dependent flow response of EUPC. The measurements were performed at 15 and 25 °C over a shear-rate range of 0.1~100 s^−1^. Apparent viscosity and shear stress were recorded to evaluate viscosity level, shear-rate dependence, and formulation-dependent flow response.

Temperature sweep tests were performed to characterize the temperature-dependent viscosity response of EUPC. The measurements were conducted from 5 to 45 °C at a heating rate of 2 °C/min, and apparent viscosity was recorded during heating.

Time sweep tests were conducted to investigate the time-dependent viscosity evolution of EUPC during the early curing stage. Apparent viscosity was continuously monitored for 3000 s at a constant shear rate of 5 s^−1^ under selected isothermal conditions. These tests were used to evaluate apparent viscosity buildup within the working period.

Three-interval thixotropy tests were conducted to assess high-shear-induced apparent viscosity reduction and apparent post-shear recovery. The test was performed sequentially using an initial low-shear interval of 1 s^−1^ for 60 s, a high-shear interval of 100 s^−1^ for 60 s, and a recovery interval of 1 s^−1^ for 900 s. These intervals were used to characterize the apparent viscosity response before high-shear disturbance, during high-shear loading, and after returning to the low-shear condition, respectively. In this study, 15 and 25 °C were selected as representative moderate ambient conditions relevant to pavement maintenance applications [4,11].

Amplitude sweep tests were conducted to determine the linear viscoelastic region (LVER) of EUPC. The measurements were performed at a fixed frequency of 1 Hz, with the strain amplitude progressively increased from 0.01% to 100%. The identified LVER was used to select the strain amplitude for subsequent oscillatory time sweep tests.

Oscillatory time sweep tests were conducted to characterize the time-dependent viscoelastic evolution of EUPC under isothermal conditions at a fixed frequency of 1 Hz. During the tests, the storage modulus G′(t), loss modulus G″(t), phase angle δ(t), and loss factor tan δ(t) were continuously recorded. The test was continued until the viscous-to-elastic transition was identified for each formulation. Under the selected oscillatory condition, the time corresponding to tan δ ≈ 1, or equivalently G′ ≈ G″ and δ ≈ 45°, was defined as the apparent gel time, tg [9,10]. This operational definition was used to compare formulation-dependent transition behavior, while recognizing that a strict critical gel point requires frequency-independent viscoelastic criteria established through multi-frequency analysis.

### 2.4. Statistical Treatment and Reproducibility

All rheological measurements were performed at least in triplicate using independently prepared samples. For tests producing continuous rheological curves, representative curves are presented when repeated measurements showed consistent curve shape, viscosity evolution sequence, and formulation-dependent response trend. The repeated tests were used to verify the reproducibility of the observed rheological behavior.

Scalar parameters extracted from repeated measurements, including initial apparent viscosity, initial viscoelastic parameters, apparent gel time, and crossover modulus, are reported as mean ± standard deviation when applicable. For 3ITT measurements, the apparent viscosity reduction ratio during the high-shear interval, *B*_η_, and the apparent recovery ratio at 900 s, *R*_η_, were extracted from representative curves selected from repeated measurements with consistent overall trends. Therefore, *B*_η_ and *R*_η_ are used as descriptive indices rather than statistically averaged material constants.

Differences among formulations were evaluated descriptively based on repeated measurements and the relative sequence of key rheological indicators obtained under identical or specified testing protocols. The rheometer geometry, testing gap, temperature-control conditions, pre-shear procedure, sample-loading procedure, and measurement programs were kept constant within each test protocol.

## 3. Results

### 3.1. Functional-Group Characteristics and Thermal Transition Behavior of EUPC Reactive Polymer Composites

The functional-group characteristics and thermal transition behavior of the cured EUPC formulations were characterized by FTIR and DSC, respectively. These results provide chemical and thermal information for understanding the formulation-dependent rheological behavior discussed in the following sections.

**Figure 2:**
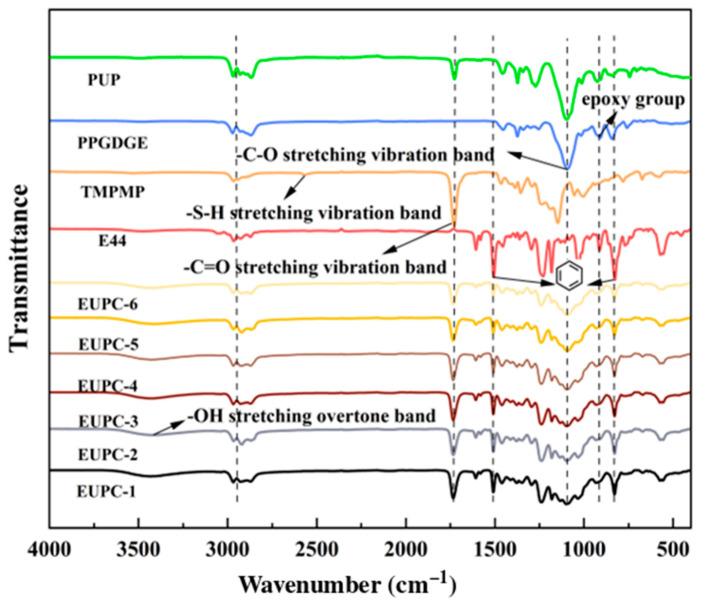
FTIR spectra of the raw materials and cured EUPC reactive polymer composites with characteristic absorption bands labeled.

Figure 2 presents the FTIR spectra of the raw materials and cured EUPC formulations. According to reported FTIR assignments [34,35,36], the main characteristic bands included the epoxy oxirane absorption near 915 cm^−1^, S–H stretching of TMPMP near 2550–2570 cm^−1^, C=O stretching of urethane/ester groups near 1720–1730 cm^−1^, C–O/C–O–C stretching at approximately 1030–1250 cm^−1^, and broad N–H/O–H stretching at 3200–3500 cm^−1^. After curing, the EUPC formulations showed broadly similar qualitative spectral features. The attenuation or absence of the S–H band and the reduction in epoxy-related absorption were consistent with the consumption of mercapto and epoxy groups, while the retained C=O and C–O/C–O–C bands indicated the presence of urethane, ester, and ether structures in the cured composites. No prominent formulation-specific new absorption band was observed.

**Figure 3:**
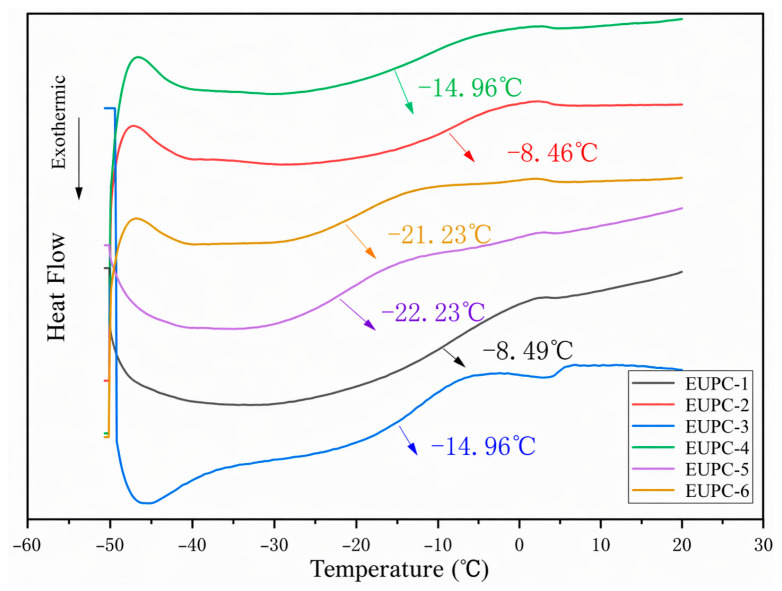
DSC thermograms and glass transition temperatures of cured EUPC reactive polymer composites.

Figure 3 shows the DSC thermograms and glass transition temperatures of the cured EUPC composites. The formulations exhibited different T_g_ values, indicating formulation-dependent thermal transition behavior. EUPC-1 and EUPC-2 showed relatively higher T_g_ values, suggesting stronger restriction of segmental mobility. In contrast, EUPC-5 and EUPC-6 showed lower T_g_ values, corresponding to greater segmental mobility and a more flexible cured polymer phase. EUPC-3 and EUPC-4 displayed intermediate T_g_ values. These results indicate that formulation adjustment affected the mobility–constraint balance of the cured composites.

### 3.2. Shear-Dependent Flow Behavior

The steady-shear response was analyzed to determine the initial processability of the uncured EUPC formulations before curing-induced structure buildup became dominant. For crack-sealing or in situ repair applications, initial viscosity is not merely a measure of fluidity; it defines the balance between crack penetration and resistance to undesired flow after placement.

**Figure 4:**
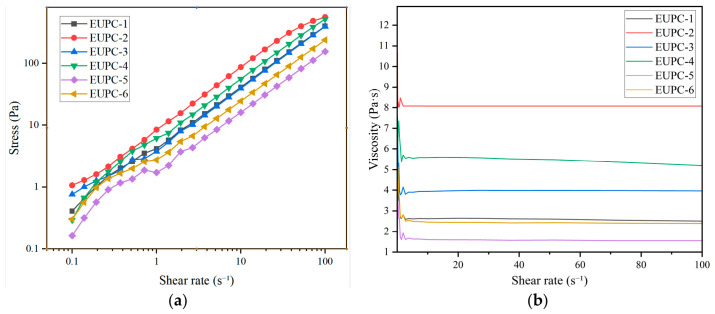
Shear-dependent flow behavior of EUPC reactive polymer composites: (**a**) shear stress versus shear rate; (**b**) apparent viscosity versus shear rate.

Figure 4 shows the shear-dependent flow behavior of the uncured EUPC formulations. As shown in Figure 4a, the shear stress increased monotonically with increasing shear rate for all formulations. When plotted on log-log coordinates, the τ–γ curves were more clearly distinguished, particularly in the low-shear-rate region. EUPC-2 and EUPC-4 exhibited higher shear stress levels over the investigated shear-rate range, indicating greater initial flow resistance, whereas EUPC-5 and EUPC-6 showed lower stress levels and thus greater initial mobility. EUPC-1 and EUPC-3 displayed intermediate responses.

The apparent viscosity curves in Figure 4b further show that the viscosity of each formulation was only weakly dependent on shear rate under the selected testing conditions. This behavior indicates that the uncured EUPC formulations were closer to near-Newtonian or weakly shear-dependent fluids than to systems with pronounced shear-thinning or shear-thickening behavior. Accordingly, the formulation-dependent differences were mainly reflected in the viscosity and stress levels, rather than in strong shear-rate-dependent flow transitions. Minor deviations in the low-shear-rate region may be associated with the higher sensitivity of stress measurement at low torque levels and the evolving state of the reactive mixtures.

These formulation-dependent differences reflect the initial processability of the uncured composites. Because steady-shear measurements only describe the initial flow response, the results were further interpreted together with time-dependent viscosity evolution, 3ITT recovery behavior, and the apparent viscous-to-elastic transition.

### 3.3. Temperature- and Time-Dependent Viscosity Evolution During Ambient Curing

Temperature and curing time were coupled in the viscosity evolution of the ambient-curing EUPC mixtures. Under the present testing conditions, increasing temperature reduced the apparent viscosity of the uncured mixtures, whereas the continued curing process led to time-dependent viscosity growth. Because these two effects occurred concurrently during measurement, the present results describe the apparent temperature- and time-dependent rheological evolution rather than quantitatively separated thermal and curing contributions.

**Figure 5:**
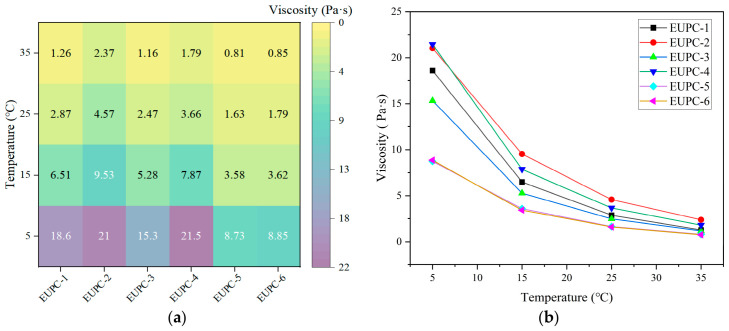
Initial viscosity and temperature sensitivity of EUPC reactive polymer composites: (**a**) heat map of initial viscosity at different temperatures; (**b**) variation in initial viscosity with temperature.

**Figure 6:**
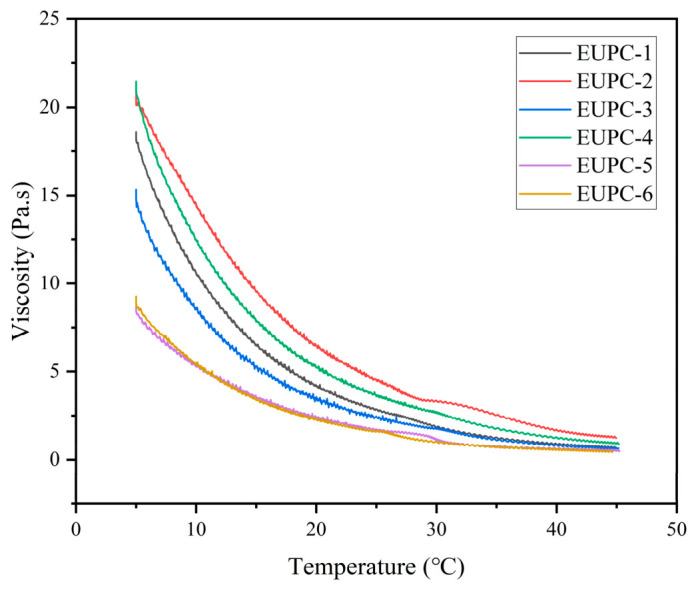
Continuous temperature sweep curves of EUPC reactive polymer composites.

Figure 5 and Figure 6 show that increasing temperature reduced the apparent viscosity of all EUPC formulations, indicating temperature-sensitive flow behavior of the uncured systems. The relative viscosity ranking among formulations remained broadly consistent over the investigated temperature range. EUPC-2 and EUPC-4 were generally located in the higher-viscosity region, whereas EUPC-5 and EUPC-6 showed lower apparent viscosity. EUPC-1 and EUPC-3 exhibited intermediate behavior.

**Figure 7:**
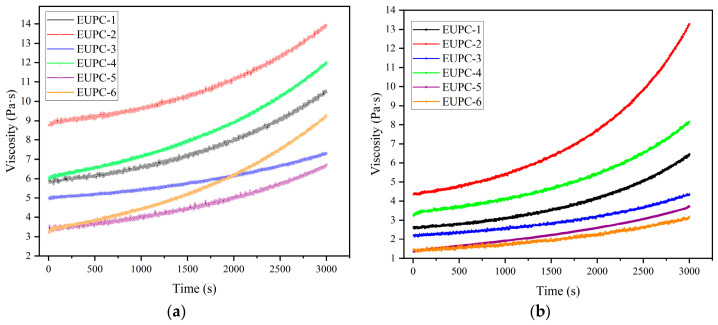
Time-dependent viscosity evolution of EUPC reactive polymer composites within the working window: (**a**) at 15 °C; (**b**) at 25 °C.

The time sweep results in Figure 7 show that the apparent viscosity increased continuously under the selected isothermal shear conditions, indicating apparent time-dependent rheological buildup in all formulations. For the ambient-curing EUPC system, this response may include both curing-related viscosity growth and shear-induced structural evolution. The temperature-dependent response was also evident: lower temperature resulted in higher initial viscosity but slower viscosity buildup, whereas higher temperature reduced the initial viscosity but accelerated the subsequent increase in apparent viscosity. Therefore, the time-dependent processability of EUPC cannot be inferred from initial viscosity alone. A formulation that appears sufficiently flowable at the beginning may show rapid viscosity buildup at higher temperature, while a formulation with higher initial viscosity at lower temperature may still retain workability because of slower time-dependent viscosity growth.

### 3.4. Shear-Induced Viscosity Reduction and Apparent Post-Shear Recovery

**Figure 8:**
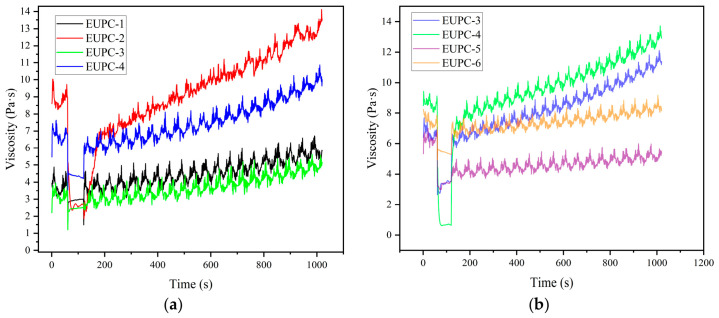
Three-interval thixotropy behavior of representative EUPC reactive polymer composites: (**a**) EUPC-1 to EUPC-4 tested at 25 °C; (**b**) EUPC-3 to EUPC-6 tested at 15 °C.

Figure 8 shows representative three-interval thixotropy test (3ITT) curves of selected EUPC formulations at 15 and 25 °C, which were selected as representative moderate ambient conditions relevant to pavement maintenance applications. During the high-shear interval, the apparent viscosity decreased for all tested formulations, reflecting high-shear-induced apparent viscosity reduction. After returning to the low-shear condition, the apparent viscosity increased again during the recovery interval. Because EUPC is an ambient-curing reactive composite, this recovery-stage response should be interpreted as apparent post-shear viscosity evolution rather than pure thixotropic recovery, as time-dependent viscosity growth may occur concurrently during the recovery interval.

To quantify the 3ITT response, the apparent viscosity reduction ratio during the high-shear interval, *B_η_*, and the apparent recovery ratio at 900 s, *R_η_*, were calculated as follows:

(1) $$B_{\eta }=\frac{\eta_{1,end}-\eta_{2,end}}{\eta_{1,end}}\times 100\%$$

(2) $$R_{\eta }=\frac{\eta_{3,end}-\eta_{2,end}}{\eta_{1,end}-\eta_{2,end}}\times 100\%$$

where *η*_1,*end*_ is the apparent viscosity at the end of the initial low-shear interval, *η*_2,*end*_ is the apparent viscosity at the end of the high-shear interval, and *η*_3,*end*_ is the apparent viscosity at the end of the recovery interval. The *B_η_* and *R_η_* values were extracted from representative 3ITT curves and are used as descriptive indices rather than statistically averaged material constants. Because viscosity continued to increase during the recovery stage, Rη should be regarded as an apparent recovery index that may include both apparent post-shear recovery and concurrent curing-related viscosity growth.

Table 3 summarizes the representative-curve-derived 3ITT parameters corresponding to the testing conditions shown in Figure 8.

As shown in Figure 8 and Table 3, all tested formulations exhibited apparent viscosity reduction during the high-shear interval, but the magnitude of reduction varied with formulation and testing temperature. At 25 °C, EUPC-2 showed a higher *B_η_* than EUPC-1, while EUPC-4 showed a higher *B_η_* than EUPC-3. At 15 °C, EUPC-4 exhibited the highest *B_η_* among the tested formulations, whereas EUPC-6 showed a lower *B_η_* than EUPC-5.

The apparent recovery response also varied among formulations. Most formulations showed *R_η_* values greater than 100%, indicating that the recovery-stage viscosity exceeded the initial low-shear viscosity. This behavior suggests that the recovery response should not be interpreted as pure thixotropic recovery alone, but may include concurrent curing-related viscosity growth. Therefore, *B_η_* and *R_η_* are used as descriptive indices of post-shear rheological evolution and should be interpreted together with time-dependent viscosity evolution and apparent gelation behavior.

### 3.5. Viscoelastic Transition and Apparent Gelation

The viscous-to-elastic transition reflects the increasing elastic contribution of EUPC mixtures during ambient curing and provides an operational indicator of rheological structure buildup. Oscillatory measurements have been widely used to characterize gelation and viscous-to-elastic transitions in reactive polymer systems [9,10]. In this study, oscillatory measurements were used to compare the formulation-dependent viscoelastic transition behavior of the uncured EUPC mixtures under the selected testing condition.

**Figure 9:**
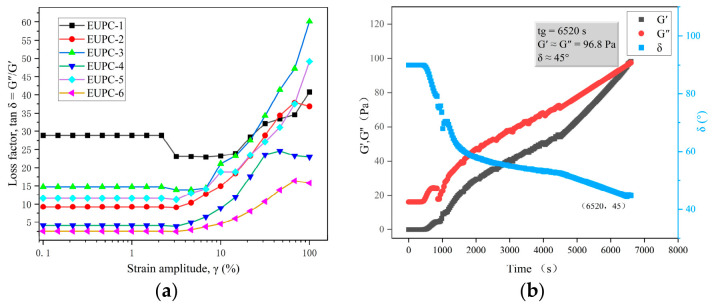
Viscoelastic response of EUPC reactive polymer composites: (**a**) strain-dependent tan δ; (**b**) time-dependent G′, G″, and δ of EUPC-6.

The amplitude sweep results in Figure 9a were used to identify the strain range for subsequent oscillatory time sweep measurements. In the low-strain region, tan δ remained relatively stable, whereas a marked increase occurred at higher strain amplitudes, indicating the onset of strain-induced nonlinearity in the viscoelastic response. Figure 9b shows the representative time-dependent evolution of G′, G″, and δ for EUPC-6 during ambient curing. The increase in G′ and G″, together with the decrease in δ, indicates a gradual transition from viscous-dominated to more elastic-dominated behavior. Based on reported rheological criteria for gel-time determination in curing epoxy systems [40], the time corresponding to G′ ≈ G″ and δ ≈ 45° was used as the apparent gel time under the selected oscillatory condition.

As summarized in Table 4, all formulations were initially dominated by viscous response, as indicated by G′ values much lower than G″ and phase angles close to 90°. With increasing curing time, the formulations reached the operational viscous-to-elastic transition at different times. The apparent gel time followed the sequence EUPC-2 < EUPC-4 < EUPC-1 < EUPC-3 < EUPC-5 < EUPC-6, indicating faster viscoelastic transition for EUPC-2 and EUPC-4, delayed transition for EUPC-5 and EUPC-6, and intermediate behavior for EUPC-1 and EUPC-3.

Compared with the large variation in apparent gel time, the crossover modulus varied within a narrower range, from 96.8 ± 1.8 to 121.5 ± 2.4 Pa. This indicates that formulation variation mainly affected the time required to reach the operational transition, rather than the modulus level at the transition point. The apparent gelation sequence therefore represents a comparative rheological response of the present EUPC formulation series under the selected oscillatory condition.

## 4. Discussion

### 4.1. Coupled Interpretation of Rheological Evolution

The rheological evolution of EUPC formulations can be interpreted as a coupled response associated with reactive-matrix composition, mineral filler incorporation, temperature-dependent mobility, shear history, and ambient curing. The FTIR results showed broadly similar qualitative functional-group characteristics among the cured formulations, whereas the DSC results revealed formulation-dependent differences in glass transition behavior. These results suggest that the observed rheological differences were not associated with fundamentally different qualitative spectral features among the cured formulations, but were more likely related to formulation-dependent segmental mobility and time-dependent rheological evolution.

This interpretation is consistent with the rheological results. EUPC-2 and EUPC-4 generally exhibited higher initial flow resistance and earlier apparent viscous-to-elastic transition, whereas EUPC-5 and EUPC-6 showed lower initial apparent viscosity and delayed apparent transition. EUPC-1 and EUPC-3 displayed intermediate behavior. Because the formulation variables varied simultaneously in the present comparative series, these trends should be regarded as whole-formulation responses rather than the isolated effect of a single component.

### 4.2. Temperature-Conditioned Processability–Structure Buildup Balance

The processability–structure buildup balance of ambient-curing EUPC formulations was evaluated by integrating the steady-shear, temperature-dependent viscosity, time-dependent viscosity, 3ITT, and oscillatory time sweep results. These measurements provide complementary information on initial flow resistance, temperature sensitivity, time-dependent viscosity buildup, post-shear response, and the operational viscous-to-elastic transition. Therefore, the rheological comparison should be based on the combined evolution of flowability and structure buildup rather than on a single viscosity value or a single apparent gel time.

Taken together, EUPC-2 and EUPC-4 were characterized by relatively higher initial apparent viscosity and earlier operational viscous-to-elastic transition, whereas EUPC-5 and EUPC-6 showed lower initial apparent viscosity and delayed transition. EUPC-1 and EUPC-3 displayed intermediate behavior. The 3ITT results further showed formulation- and temperature-dependent apparent viscosity reduction and recovery-stage viscosity evolution. Overall, these rheological differences should be interpreted as temperature-conditioned whole-formulation responses under the selected testing protocols.

### 4.3. Implications, Limitations, and Future Work

The present study provides a laboratory-scale rheological comparison of ambient-curing EUPC formulations by integrating initial flow resistance, temperature-dependent viscosity, time-dependent viscosity buildup, post-shear response, and operational viscous-to-elastic transition. The results describe the processability–structure buildup balance of the tested formulations under the selected rheological protocols and provide a basis for subsequent performance-oriented evaluation, although direct application-performance ranking requires further validation.

Future work should further correlate these rheological indicators with application-related performance, including penetration ability, interfacial bonding, deformation stability, curing-condition adaptability, and durability.

## 5. Conclusions

In this study, the rheological evolution and viscoelastic transition of ambient-curing epoxy–urethane reactive polymer composites were investigated. The main conclusions are as follows:

(1) The cured EUPC formulations showed broadly similar qualitative FTIR features, while DSC results revealed formulation-dependent glass transition behavior. These results indicate that the cured formulations had comparable qualitative functional-group characteristics but different segmental mobility.
(2) The uncured EUPC formulations exhibited different initial flow resistance and temperature-sensitive viscosity behavior. At 25 °C, EUPC-2 and EUPC-4 showed relatively higher initial apparent viscosity, whereas EUPC-5 and EUPC-6 showed lower values.
(3) Time-dependent measurements showed continuous apparent viscosity buildup during ambient curing. The results indicate that EUPC processability depends on both initial flowability and curing-time-dependent rheological evolution.
(4) The 3ITT results showed high-shear-induced apparent viscosity reduction followed by recovery-stage viscosity evolution. The apparent viscosity reduction ratio, *B_η_*, and apparent recovery ratio, *R_η_*, provide descriptive indices for comparing post-shear rheological responses. Because the recovery-stage viscosity increase may include concurrent curing-related contributions, *R_η_* should be interpreted as an apparent recovery index rather than as a purely thixotropic recovery parameter.
(5) Oscillatory measurements revealed formulation-dependent operational viscous-to-elastic transition behavior. Under the selected oscillatory condition, the apparent gel time followed the sequence EUPC-2 < EUPC-4 < EUPC-1 < EUPC-3 < EUPC-5 < EUPC-6. This sequence should be interpreted as a comparative whole-formulation response rather than as the isolated effect of a single compositional variable.

Overall, initial flow resistance, temperature sensitivity, time-dependent viscosity buildup, post-shear response, and operational viscous-to-elastic transition should be considered together when comparing ambient-curing EUPC formulations.

## Figures and Tables

**Table 1:** Raw material information and supplier-provided specifications.

Function	Raw Material	Properties	Results
Resin matrix	DGEBA (E-44) (Nantong Xingchen Synthetic Material Co., Ltd., Nantong, China)	Viscosity (mPa·s, 25 °C)	18,000–25,000
		Density (g/mL, 25 °C)	1.17
		Epoxy Value (eq/100 g)	0.42–0.46
Toughener	PUP (Shandong Yiborun New Materials Technology Co., Ltd., Binzhou, China)	Viscosity (mPa·s, 80 °C)	250–300
		Density (g/mL, 25 °C)	1.16
Curing agent	TMPMP (Guangzhou Geling New Materials Co., Ltd., Guangzhou, China)	Viscosity (mPa·s, 25 °C)	135–165
		Density (g/mL, 25 °C)	1.15–1.30
Diluent	PPGDGE (Guangzhou Yinghong Chemical Co., Ltd., Guangzhou, China)	Viscosity (mPa·s, 25 °C)	40–70
		Density (g/mL, 25 °C)	1.05
		Epoxy Value (eq/100 g)	0.28–0.36
Catalyst	DMP-30 (Guangzhou Geling New Materials Co., Ltd., Guangzhou, China)	Viscosity (mPa·s, 25 °C)	80–150
		Density (g/mL, 25 °C)	0.98
		Amine Value, mgKOH/g	600 ± 20
Filler	PCC (Hebei Hongyao Mineral Products Processing Co., Ltd., Shijiazhuang, China)	Particle Size(D_50_, μm)	2.5–3.5
		Bulk Density (g/cm^3^, 25 °C)	1.2
		CaCO_3_ Purity (%)	≥98.5

Note: The listed properties were obtained from supplier-provided technical specifications. These values are used only for raw-material description and not for quantitative rheological analysis in this study.

**Table 2:** Formulation proportions of EUPC reactive polymer composites.

Specimen	DGEBA	PUP	PPGDGE	TMPMP	DMP-30	PCC
EUPC-1	100	25	80	74.1	2.8	88.0
EUPC-2	100	25	80	76.7	2.8	112.0
EUPC-3	100	25	100	80.2	3.0	92.4
EUPC-4	100	25	100	83.7	3.0	123.1
EUPC-5	100	25	140	86.5	3.6	108.1
EUPC-6	100	25	140	94.0	3.6	145.0

Note: All values are expressed as parts by mass, with E44 fixed at 100 parts.

**Table 3:** Representative-curve-derived 3ITT parameters of EUPC reactive polymer composites at 15 and 25 °C.

Specimen	Temperature/°C	*η*_1,*end*_/Pa·s	*η*_2,*end*_/Pa·s	*η*_3,*end*_/Pa·s	*B_η_*/%	*R_η_*/%
EUPC-1	25	3.95	3.00	5.61	24.0	275.2
EUPC-2	25	9.13	2.70	13.60	70.4	169.5
EUPC-3	25	3.47	2.50	5.08	28.0	266.6
EUPC-4	25	6.88	4.23	9.96	38.5	216.6
EUPC-3	15	6.73	3.51	11.35	47.9	243.2
EUPC-4	15	8.79	0.66	13.14	92.4	153.5
EUPC-5	15	6.61	3.50	5.38	47.0	60.4
EUPC-6	15	7.53	5.30	8.33	29.7	135.7

**Table 4:** Viscoelastic parameters associated with the apparent gelation of EUPC reactive polymer composites.

Specimen	Initial G′ (Pa)	Initial G″ (Pa)	Initial δ (°)	Apparent Gel Time, tg (s)	G′ ≈ G″ at tg (Pa)
EUPC-1	1.20 ± 0.06	19.00 ± 0.03	86.39 ± 0.18	4320 ± 20	104.5 ± 2.1
EUPC-2	2.50 ± 0.11	26.10 ± 0.05	84.53 ± 0.24	2980 ± 14	121.5 ± 2.4
EUPC-3	0.90 ± 0.01	17.50 ± 0.01	87.06 ± 0.03	4770 ± 20	99.0 ± 1.9
EUPC-4	1.80 ± 0.02	22.00 ± 0.04	85.32 ± 0.05	3775 ± 6	112.3 ± 2.2
EUPC-5	0.60 ± 0.01	16.80 ± 0.01	87.95 ± 0.03	5310 ± 20	97.2 ± 1.8
EUPC-6	0.50 ± 0.01	16.20 ± 0.02	88.23 ± 0.02	6520 ± 20	96.8 ± 1.8

Note: Values are reported as mean ± standard deviation based on three repeated measurements.

## Data Availability

The original contributions presented in this study are included in the article. Further inquiries can be directed to the corresponding author.

## Abbreviations

The following abbreviations are used in this manuscript:

EUPC	Ambient-curing epoxy–urethane/precipitated calcium carbonate reactive polymer composite
E44	E44 epoxy resin
PUP	Isocyanate-terminated polyether polyurethane prepolymer
TMPMP	Trimethylolpropane tris(3-mercaptopropionate)
PPGDGE	Poly(propylene glycol) diglycidyl ether
DMP-30	2,4,6-Tris(dimethylaminomethyl)phenol
PCC	Precipitated calcium carbonate
FTIR	Fourier transform infrared spectroscopy
DSC	Differential scanning calorimetry
Tg	Glass transition temperature
3ITT	Three-interval thixotropy test
LVER	Linear viscoelastic region
G′	Storage modulus
G″	Loss modulus
δ	Phase angle
tan δ	Loss factor
*tg*	Apparent gel time
*η*	Apparent viscosity
*R_η_*	Apparent viscosity recovery ratio
